# Path taken by morbidly obese people in search of bariatric surgery in the public health system*

**DOI:** 10.1590/1518-8345.3579.3294

**Published:** 2020-07-15

**Authors:** Claudete Aparecida Conz, Maria Cristina Pinto de Jesus, Estela Kortchmar, Vanessa Augusta Souza Braga, Renata Evangelista Tavares Machado, Miriam Aparecida Barbosa Merighi

**Affiliations:** 1 Universidade de São Paulo, Escola de Enfermagem, São Paulo, SP, Brazil.; 2 Scholarship holder at the Coordenação de Aperfeiçoamento de Pessoal de Nível Superior (CAPES), Brazil.; 3 Universidade Federal de Juiz de Fora, Faculdade de Enfermagem, Juiz de Fora, MG, Brazil.; 4 Scholarship holder at the Conselho Nacional de Desenvolvimento Científico e Tecnológico (CNPq), Brazil.

**Keywords:** Obesity, Bariatric Surgery, Health Services, Comprehensive Health Care, Nursing, Qualitative Research, Obesidade, Cirurgia Bariátrica, Serviços de Saúde, Assistência Integral à Saúde, Enfermagem, Pesquisa Qualitativa, Obesidad, Cirugía Bariátrica, Servicios de Salud, Atención Integral de Salud, Enfermería, Investigación Cualitativa

## Abstract

**Objective:**

to understand the path taken in the public health system by people with morbid obesity in the search for bariatric surgery.

**Method:**

qualitative research based on the social phenomenology of Alfred Schütz, with 17 hospitalized morbidly obese people, with a scheduled date for bariatric surgery. The phenomenological interview with open questions was used and the statements were analyzed in the light of the theoretical-methodological framework and literature related to the theme.

**Results:**

the participants were able to schedule bariatric surgery by referring friends, family and public people. The waiting list for the procedure generated anguish and anxiety due to fear of surgery, weight gain, risk of worsening health and physical limitations, but it helped prepare for its performance. The experience lived in the search for bariatric surgery led these people to want continuity of care in the Basic Health Unit, after the surgery, by professionals trained to meet their needs.

**Conclusion:**

the aspects inscribed in the path of people in search of bariatric surgery signal the need to strengthen the assistance-related flows of the public health system and to invest in professional training to reduce the social inequalities in access to bariatric surgery and increased quality of services.

## Abstract (pt)

**Objetivo:**

compreender o caminho percorrido no sistema público de saúde pela pessoa com obesidade mórbida em busca da cirurgia bariátrica.

**Método:**

pesquisa qualitativa fundamentada na fenomenologia social de Alfred Schütz, com 17 pessoas obesas mórbidas hospitalizadas, com data agendada para a cirurgia bariátrica. Utilizou-se a entrevista fenomenológica com questões abertas e os depoimentos foram analisados à luz do referencial teórico-metodológico e literatura referente ao tema.

**Resultados:**

os participantes conseguiram agendamento para a cirurgia bariátrica por meio da indicação de amigos, familiares e pessoas públicas. A fila de espera pelo procedimento gerou angústia e ansiedade devido ao medo da cirurgia, aumento do peso, risco de agravamento da saúde e das limitações físicas, mas auxiliou na preparação para sua realização. A experiência vivida na busca pela cirurgia bariátrica levou essas pessoas a desejarem a continuidade da assistência na Unidade Básica de Saúde, após a cirurgia, por profissionais capacitados para atender as suas necessidades.

**Conclusão:**

os aspectos inscritos no caminho de pessoas em busca de cirurgia bariátrica sinalizam a necessidade de fortalecimento dos fluxos assistenciais do sistema público de saúde e de investimento na capacitação profissional para redução das desigualdades no acesso à cirurgia bariátrica e incremento da qualidade dos serviços.

## Abstract (es)

**Objetivo:**

comprender el camino que recorre la persona con obesidad mórbida en el sistema de salud pública en la búsqueda de cirugía bariátrica.

**Método:**

investigación cualitativa basada en la fenomenología social de Alfred Schütz, con 17 personas internadas con obesidad mórbida, con fecha programada para cirugía bariátrica. Se utilizó la entrevista fenomenológica con preguntas abiertas y las declaraciones se analizaron a la luz del marco teórico-metodológico y la literatura relacionada con el tema.

**Resultados:**

los participantes pudieron programar la cirugía bariátrica gracias a la recomendación de amigos, familiares y personas públicas. La lista de espera para el procedimiento generó angustia y ansiedad debido a factores como el miedo a la cirugía, aumento de peso, riesgo de empeorar el estado de salud y limitaciones físicas, pero ayudó a los participantes a prepararse para su realización. La experiencia vivida en la búsqueda de cirugía bariátrica llevó a estas personas a pretender la continuidad de la atención por profesionales capacitados para satisfacer sus necesidades en la Unidad Básica de Salud, después de la cirugía.

**Conclusión:**

los vaivenes que se suscitan en el camino de las personas en búsqueda de cirugía bariátrica señalan la necesidad de fortalecer los flujos de atención del sistema de salud pública e invertir en capacitación profesional para reducir las desigualdades en el acceso a la cirugía bariátrica y aumentar la calidad de los servicios prestados.

## Introduction

Obesity is a worldwide public health problem that requires the organization of the health system to serve those who experience this condition. It is a complex, multicausal disease, rooted in the sedentary nature of modern life, provided by the offer of more widely available and accessible foods, by the change in the nature and composition of diets and stimuli for the consumption of multi-processed foods. Excessive weight gain is multifactorial, so it should be considered in a multidimensional way in actions that enhance the aspects involved in the search for obesity treatment^(^ 1 ^)^.

Research carried out in 186 countries showed that the number of obese people in the world has grown six times in the last four decades, from 105 million overweight individuals in 1975 to 641 million in 2014. If the world population continue to gain weight at the current rate, about a fifth of the world’s people will be overweight in less than ten years. In 2025, the prevalence of global obesity will reach 18% in men and exceed 21% in women, and obesity grade III or more will exceed 6% in men and 9% in women^(^ 2 ^)^.

Among the possibilities for treating obesity is bariatric surgery, especially in the case of severely obese patients, with effective and lasting results in controlling weight and associated diseases^(^ 3 ^)^. In order to perform this surgical procedure in the context of the Brazilian Unified Health System (SUS), policies and programs recommend that the initial access be made preferably at the level of Primary Health Care (PHC), through the Basic Health Units (UBSs) that order the assistance-related flow in services with different technological densities^(^ 4 ^)^.

In order to favor the path taken by people with obesity in the health system, evidence pointed out that it is necessary to establish a strong communication network between care providers, to share information, to respect regional beliefs and cultures^(^ 5 ^)^, use tools available for clinical evaluation and referral to specialists, when necessary^(^ 6 ^)^, and promote multidisciplinary action centered on the patient^(^ 7 ^)^.

However, during the journey in search of bariatric surgery, obese people may encounter obstacles in health services that negatively impact the continuity of their treatment, which may hinder the management of the condition and increase the risk for comorbidities. A study carried out in Canada revealed that the majority of doctors working in the PHC have already referred patients to undergo bariatric surgery, however they realize the scarcity of resources and training to manage the set of health cares before and after this procedure^(^ 8 ^)^. This situation interferes with the referral and the path taken in search for the surgery, since there are limitations in directing this flow of care in the health system.

Obese people seek care in the public health system to resolve issues related to excess body weight based on common sense knowledge, influenced by subjective and individual or collective constructions and interpretations, which are not always adequate to the recommended flows. This causes a succession of events and decision making that lead these people to find different ways to build the path with a view to solving their health problems^(^ 9 ^)^.

Understanding the reality experienced by people with morbid obesity on the path taken in the Brazilian public health system may provide evidence for the development of effective policies, with the potential to bridge the gap between what is experienced and the received care. In this perspective, the construction of this research was made by the following question: How people with morbid obesity perceive the path taken in the public health system, from entering the health system to admission to bariatric surgery? Thus, the objective of this study is to understand the path taken in the public health system by people with morbid obesity in the search for bariatric surgery.

## Method

It is a qualitative research, anchored in Alfred Schütz’s social phenomenology. This framework recommends that the understanding of subjective experience is based on people’s daily lives, permeated by the relationships that constitute the social world. Man expresses his experiences that were experienced in the past and present (reasons why) and projects the achievement of goals (reasons for) based on the intersubjectivity that allows him to identify and be identified within a given social group. Intersubjectivity enables interaction in a social context characterized by the physical, sociocultural environment in which you experience everyday situations^(^ 10 ^)^.

The study was carried out in a public hospital in the city of São Paulo, Brazil, considered one of the largest hospital complexes in Latin America. This has a referral center for the treatment of obese people and bariatric surgery procedures, has a multidisciplinary care clinic and a rear hospital, where patients with a body mass index above 40 kg/m^2^ remain in follow-up for six months to weight reduction and reduction of the surgical risks.

The study included people with morbid obesity, over 18 years of age, who sought treatment for obesity in the public health system and who met the criteria for performing bariatric surgery. We sought out patients who were admitted to the medical-surgical clinic of the Bariatric and Metabolic Surgery Unit of the hospital where the study was conducted to schedule bariatric surgery, and the selection of participants was therefore intentional. It is noteworthy that, in this Inpatient Unit, those who have surgery scheduled are hospitalized for seven to ten days before the procedure and are prepared by the health team, aiming at maintaining food reeducation and weight loss after surgery.

Before approaching the patients, the main researcher, who was in the process of obtaining a PhD, established an interaction relationship with all possible participants, in order to interpret the meaning of spoken and unspoken expressions at the time of data collection. After clarifying the research, an invitation to participate was made.

With the acceptance of the participants, a day and time of better convenience was scheduled for each one to participate in the interview, and everyone was informed about the research objectives, ethical aspects involved and the need to sign the Free and Informed Consent Form. 17 interviews were conducted and no one refused or gave up participating in the study.

For data collection, phenomenological interview conducted between January and April 2017 was used, guided by the questions: Considering your experience with obesity and the fact that you have scheduled bariatric surgery, how was your search for this surgical procedure in the public health system? Which paths did you take? How do you expect to continue your treatment after the surgery in the public health system? The phenomenological interview was adopted because it allows the interviewee to encounter the phenomenon experienced and has peculiarities that should be considered by the researcher, such as the refraining from moral judgments and prejudices, so that it can constitute the basis of the phenomenological analysis. The guiding questions allow people to express what is in his consciousness^(^ 11 ^)^.

The interviews were conducted in a private, reserved room, in the medical-surgical clinic unit of the hospital and were recorded on audio after the participants’ permission. The duration of the interviews lasted 30 to 50 minutes. The 17 testimonies obtained were included in the research, considering the wealth of meanings and, therefore, there were no sample losses.

Data collection ended when the significant content of the data was reached and no new themes emerged, indicating that the objective of the study was achieved and the research questions answered^(^ 12 ^)^. To guarantee the anonymity of the participants, the interviews were identified by the letter P, initial of the word “Participant”, followed by Arabic numerals corresponding to the order of the interviews (P1 to P17).

Ethical recommendations on research with human beings were complied with, as recommended by the National Health Council in the resolution No. 466/2012. This study was submitted to the Research Ethics Committee of the School of Nursing of the University of São Paulo and the Hospital das Clínicas of the Faculty of Medicine of the University of São Paulo, obtaining favorable opinions No. 1.915.867 and No. 1.956.427, respectively.

For the organization, categorization and analysis of the interviews, the steps proposed in a study highlights the main conceptions of Alfred Schütz’s social phenomenology and his contribution to care and research in Nursing^(^ 13 ^)^. These steps consisted of transcription, in-depth reading of the testimonies to obtain the past and present experiences of people with morbid obesity in search of bariatric surgery in the public health system (reasons why), as well as their expectations related to care after the surgical procedure (reasons for). The set of reasons why and reasons for identified allowed the construction of the study’s thematic categories. These were discussed in the light of the theoretical-methodological framework and thematic of the study.

The rigor and credibility were maintained during the interviews, transcriptions, analysis, construction of the categories and discussion of the data. The transcription of the statements preserved the reliability, taking into account only what was verbalized. The interviews, transcriptions and analysis were done by the main researcher in order to capture feelings, perceptions and experiences in as much detail as possible.

The survey followed the steps recommended by the Consolidated Criteria for Reporting a Qualitative Survey (COREQ)^(^ 14 ^)^.

## Results

The study consisted of four men and 13 women, aged between 18 and 70 years old, with an education level going from elementary school to higher education. The range of body mass index was 40.44 to 80.89 kg/m^2^ and the waiting time for bariatric surgery ranged from 15 days to nine years.

Based on Alfred Schütz’s social phenomenology, three thematic categories were attained, namely: the path taken in the search for bariatric surgery; the wait for bariatric surgery; need for continuity of care after bariatric surgery. The first and second categories are related to the reasons “why” the experience of people with morbid obesity in the journey through the public health system in search of bariatric surgery, representing the past and the present of this experience. The third category refers to the participants’ expectations regarding care in the health system and is related to the reasons “for”.

### The path taken in the search for bariatric surgery

Most of the patients referred to going through different paths until reaching bariatric surgery. Such paths are not those considered in the flow recommended by the Unified Health System: *a colleague told me that here they were doing an evaluation for the surgery. I came and stood in line for nine years. During all this time, I went through several doctors and a psychologist (P3); When I started to put on weight, I went to the health center, but they did nothing to help me lose weight, they just told me I was chubby and that I had to diet* [...] *I passed the endocrine, I did the interview with the nurse, but I didn’t have a follow-up for obese people* [...] *I tried diet and exercise, but always ended up putting on weight again.* [...] *I went to the obesity service here [hospital] because there is a queue for employees in surgery (P5); I have a friend undergoing treatment here at the hospital. She talked to her doctor about me* [...] *I came and started treating rheumatism. Then he referred me to the psychiatrist, who saw that I was overweight and referred me to bariatric surgery (P6); I went to another institution and they did not accept me because they did not serve people outside their region. So I came here alone, I asked where I should apply for the bariatric surgery (P10);* [...] *I arrived here at the hospital, I signed up, I went through the whole obesity group.* [...] *due to my weight and my young age, they already put me right in the line (P11); My niece worked with the Governor. She wrote a letter to the Secretary of State, who called securing a vacancy for me (P12); In the place where my mother lived, there is a university, with a clinic that serves the population, and it was through the nurse there that I met the Hospital.* [...] *the nurse is an employee here and introduced me to the doctor who put me in the line in 2014 (P13);.. my nephew is the son of a bariatric surgeon and works as a psychologist here.* [...] *as my wife does treatment here, I always come with her, I decided that I wanted to operate* [...] *so I signed up* [...] *I went in consultation with the doctor and got in line. Fifteen days later, I was called (P14);* [...] *I made the follow-up at the health center with psychotherapy, a psychologist and an occupational therapist, and they did not refer me.* [...] *I also took medicine and I was unable to lose weight* [...] *in 2009, I came looking for the service alone. After I signed up, I was followed up at the hospital and at the health center (P15).*

Only one of the participants reported having followed the flow recommended by the Unified Health System to reach the bariatric surgery: [...] *I went to the basic health unit and the general practitioner referred me to the specialty medical clinic. There I was directed to the endocrinologist, who calculated my body mass index and said that the only thing that would solve it was the bariatric surgery* [...] *so I came and registered at the counter (P16).*

### The wait for bariatric surgery

The participants reported that the waiting list for bariatric surgery generated anguish and anxiety due to fear of the surgical procedure, weight gain, risk of worsening health and physical limitations: [...] *I’ve been in the line for more than six years. In that time, what I felt was a great anxiety. Each time my weight increased more and more (P1); Staying in the line was distressing. When I started to go to consultations, I was very courageous, but, as time went by, I wanted to give up many times.* [...] *I have several friends who are diabetic and are losing their foot, finger, nose because of the diabetes. But, the possibility of the surgery gives me a new spirit (P2);* [...] *I stayed in the line for three months, but waiting is a struggle, an anxiety, because I didn’t know if I would have another chance. I would climb on the scale and only see the weight increasing (P4);* [...] *it is distressing to wait because there is that thing of wanting to lose weight and not being able to.* [...] *after the last time they called me to do an evaluation, I thought it would be the surgery right away, but it’s not like that, it still takes a while, and this is anguish (P5);* [...] *every year I stay in the line, I get worse because I get fatter and older (P6);...] I was very afraid of the surgery.* [...] *at the same time that I wanted to, I was afraid and kept thinking: If they call me, what will i do? (P7); In these seven years controlling me, I didn’t get fat, I didn’t lose weight, I was like this, stagnant. I went to the line with 139 kilos.* [...] *it sometimes crossed my mind to give up, try something else.* [...] *there are many different sensations and emotions. I often got distressed, sad* [...] *(P8);* [...] *during the time in line, I felt it got worse every year. I got to the point where I couldn’t do everyday things, like sweeping the house, showering and tying my shoe laces (P13);* [...] *the wait was a depressing, distressing process(P16);* [...] *I stood in line for seven years.* [...] *I thought they would never call me.* [...] *when I came for the consultation and the doctor told me my turn was coming, I was 104 kilos and referred me to the psychologist (P17).*

According to one of the participants, this time favored the maturation necessary to perform bariatric surgery: [...] *to stay in the line all this time was good because I matured. If I had operated in six months, I would have done nonsense (P15).*

### Need for continuity of care after the bariatric surgery

The participants hope to continue to be followed up at the Basic Health Unit after bariatric surgery, but wish that there are trained professionals to enable them to meet their needs: [...] *I hope to continue doing follow-up here [hospital], but after the surgery, I will have to stay at the health center* [...] *I hope the post has physical structure and professionals prepared to receive me and accompany me (P1);* [...] *the health center could have a copy of my medical record and know about my case to support me* [...] *(P5);* [...] *after the surgery, I will need follow-up so as not to put on weight again, because it’s not just about having the surgery.* [...] *I wish there was a professional who was a reference for me, which I could trust.* [...] *someone from the health center, to accompany me, to help me continue (P7);* [...] *I think that health center professionals must be prepared.* [...] *have different opening hours for treating the people so that they do not reach the stage of major obesity as I reached.* [...] *(P8);* [...] *it would be very good if I had a follow-up with the nutritionist associated with the psychologist to help me not only with the surgery, but with follow-up at least for the next few years and, if possible, for the rest of my life.* [...] *if there were treatment groups for obese people at the health centers, incentive campaigns, going after people, not waiting for them to look for the center, doing a census in the neighborhood to find out how many are obese, because obese people in the neighborhood know each other, promote a group and bring these people together to answer questions, to talk about diets and physical exercise (P9);* [...] *after I have the surgery, I hope to continue to be attended by all the professionals who have already attended me.* [...] *I started* treatment at the health center and continued at the hospital [...] *so I hope it continues because the treatment is good, the professionals are always willing to help us with anything and are competent (P11);* [...] *I hope to be able to return to the basic unit near my home and be accompanied by them too.* [...] *I hope to be accompanied by the professionals there, I think it’s important (P12).*

## Discussion

In the context of “reasons why”, the statements refer to the experience of people with morbid obesity throughout their life with this condition, especially in the path taken in search of bariatric surgery in the public health system.

In this perspective, most participants reported not having received the referral from the professionals of the Basic Health Units to perform bariatric surgery and who had access to the procedure by referring friends, family and public persons. Thus, PHC level showed gaps regarding the flow recommended by the public health system and access to care with greater technological density. The integration between services also presents weaknesses in some countries that have a well established PHC level, such as the United Kingdom and the Netherlands^(^ 15 ^)^.

The importance of strengthening PHC is emphasized, expanding and qualifying the scope of actions provided through training of professionals from multidisciplinary teams, intersectoriality, the use of protocols and tools for the effective management of obesity^(^ 16 ^)^. The strengthening of this level of health care will enable the appropriate and timely referral of people with obesity to secondary and tertiary care services.

Scientific research presents other difficulties experienced by people with obesity in search of bariatric surgery. In Canada, a study showed that less than a third of people looking for bariatric surgery received guidance on how to proceed with the specifics of the surgery. In most cases, information was acquired through contact with patients who had already undergone surgery, friends, family and through the internet^(^ 17 ^)^.

The content of the interviews showed that there was no referral to bariatric surgery based on the management of obesity by PHC professionals, which highlights gaps in the management of obesity at this level of care and limits longitudinal care to obese people.

A research conducted with 31 PHC professionals in the United States showed that the main difficulties in assisting obese people were the lack of interprofessional integration and limitations in the concept of obesity as a chronic condition, impairing the management and referral of cases^(^ 18 ^)^. A study carried out with 24 physicians in Australia pointed out that the majority of referrals for patients with obesity occurred without systematic evidence of the need for surgical intervention, which are based on their own professional attitudes and experiences, as well as on the motivation of patients^(^ 19 ^)^.

The path taken by people with morbid obesity in search of bariatric surgery points to the individuality of their experience and at the same time signals the experience of the group of patients who seek this procedure. That’s because, according to the social phenomenology of Alfred Schütz, although individual, the human experience is situated in the context of social relations experienced in everyday life. This context shows the subjectivity of each person, whose purposes and objectives are rooted in the past and present of their life history. However, the intersubjectivity being typical of the social context makes these purposes and objectives common, giving rise to a social meaning^(^ 10 ^)^.

According to the interviewees, remaining in the waiting list for a long time generated feelings of anxiety and anguish motivated by fear of surgery, weight gain and worsening health. Similar results were found in a survey conducted in Australia with 17 people waiting for bariatric surgery. The participants considered the waiting for the procedure to be an emotional challenging period (frustrating, depressing, stressful) and causing weight gain and deterioration of physical health, with the development of comorbidities or worsening mobility and depression^(^ 20 ^)^.

Considering that those who wait in the waiting list for bariatric surgery have comorbidities associated with obesity, the delay in carrying out the procedure can aggravate pre-existing health conditions. Research carried out in Spain showed that delays in carrying out the procedure generated clinical repercussions for patients. Among the health professionals participating in the research, 46.2% reported cases of patients who, in the last five years, suffered cardiovascular events with sequelae while waiting for surgery, and 21.2% cited the occurrence of fatal cardiovascular events in this period^(^ 21 ^)^.

A study carried out in Canada revealed that the prolonged waiting time for bariatric surgery and the high level of commitment required in the mandatory preoperative program were part of the set of barriers that motivated the search for bariatric surgery in another country^(^ 22 ^)^.

It is noteworthy that those who wait for bariatric surgery suffer impacts on their quality of life, generated by the high degree of obesity. Research carried out with obese Iranian patients who decided to undergo bariatric surgery identified that such people reported pain, fatigue, impaired mobility, difficulty sleeping, fear of associated diseases, poor self-care and psychological problems, which motivated them to perform the procedure^(^ 23 ^)^.

It calls attention, the fact that, for some people, the long waiting time for bariatric surgery helped to reflect on the importance of maturation in view of the need to undergo the surgical procedure. Professional actions aimed at monitoring these people, preparing them to adopt healthy lifestyle habits, as well as maintaining care after surgery, can help these patients.

With regard to the issue of severe obesity, the evaluation and selection of patients, candidates for bariatric surgery, require some preparation time. According to social phenomenology, this time is existential and is experienced individually, although located in a context of social interactions. The intersubjective world is made up of interpersonal relationships that accumulate throughout life and are arranged in a specific time and geographic space. These relationships may or may not be reciprocal, but somehow they influence people’s way of acting and reacting to a particular situation^(^ 10 ^)^.

The participants of the present study expressed the expectation (reasons for) to be followed up after being submitted to bariatric surgery in order to meet their needs and achieve the desired results for the postoperative period, demanding qualified human resources in the Basic Health Units.

The interviewees refer to the importance of monitoring at the Basic Health Units of people who have undergone bariatric surgery and those who have not reached morbid obesity, in order to prevent the disease from advancing. These statements reinforce what is recommended by Brazilian health policies regarding the care of people with obesity, with PHC as a level of care capable of solving most of the population’s health needs and as a care coordinator in the Health Care Network.

Care continuity after the bariatric surgery is an important indicator in verifying the achievement of established goals, in addition to providing conditions to assess whether patients’ expectations have been met. Research developed with 18 adults who underwent bariatric surgery in England identified that the perceptions regarding the care received after the surgical procedure were not evaluated by health service professionals, which generated in the participants frustration and a feeling of not solving problems^(^ 24 ^)^.

The treatment of obesity must consist of an integrated and multidisciplinary approach, so that the PHC professionals can execute action plans with the patients. To this end, it is necessary to train the health team to deal with the psychological, behavioral and physical aspects of obese people, as well as the risks involved in this disease and the types of treatment that can be offered. However, knowledge alone is not enough to meet the demands of this population. In order for the care to be effectuated, financial and structural resources are required, as well as adequate dimensioning of professionals in various types of knowledge^(^ 18 ^,^ 25 ^)^.

It is emphasized that health professionals need to incorporate patient incentive strategies in their practice so that they can manage their own health, through guidance on the impacts of overweight and bariatric surgery in the medium and long term, providing increased co-responsibility for the treatment and the change in the lifestyle^(^ 26 ^)^. The effectiveness of these guidelines can be enhanced if professionals consider the patient’s experiences, through a horizontal and cooperative relationship that takes place in everyday social interaction, according to the assumptions of Alfred Schütz’s social phenomenology^(^ 10 ^)^.

The weight control of people with obesity can be achieved with the work of health professionals, especially in PHC, through the adoption of strategies that provide guidance and support to people with excess weight. In this regard, a study conducted in Salvador, Bahia, Brazil, showed that remote monitoring by nurses through telephone calls based on advice for adopting healthy habits was able to reduce weight and body mass index in obese women^(^ 27 ^)^.

The involvement between the social actors who were part of the path taken by the participants in search of bariatric surgery permeated the intersubjective interactions and enabled people with obesity to perceive the flaws and potentialities of this path. It should be noted that the expectation of continuing to monitor health needs is a potential to be achieved.

According to the social phenomenology, the life world is the social reality that can be modified by the people who act in it, based on their own intentionalities and purposes, modifying the structure in which they are located. Projects and purposes are elements that form the relational system among those who share the same time and space in the social world. In this way, any choice will be based on what has already been experienced and becomes sensitive to questioning about the situation experienced. This allows people to rebuild new choices and new life plans at the individual and social levels^(^ 10 ^)^.

This study has as a limitation the unique experience of people with obesity in the search for bariatric surgery accompanied in a metropolis located in the Southeast of Brazil. Different realities may allow other findings due to the structuring of health services, which may differ in other regions of Brazil and the world, preventing the generalization of the results.

The present study contributes to the advancement of scientific knowledge by identifying aspects registered in the path of people with obesity in search of bariatric surgery in the public health system, signaling the need to strengthen the Health Care Network. Thus, the effectiveness of the recommended care flow may contribute to comprehensive care for obese people. These results may stimulate the development of strategies with this population and new research to support evidence-based practice.

It is recommended to invest in the referral and counter-referral flows at different levels of the health system, consolidated in public policies, which may reduce inequalities in access to bariatric surgery, and in the improvement of professional training programs to equip multidisciplinary teams that work together to people with obesity, increasing the quality of the health services.

## Conclusion

The understanding of the path taken in the public health system in Brazil by people with morbid obesity, from the perspective of Alfred Schütz’s social phenomenology, revealed gaps that require investment by public managers regarding the effectiveness of referral and counter-referral flows at different levels health system to serve this public. The recommended flow and access to care with a higher technological density do not meet the needs of these people, imposing the search for referrals from friends, family and public people to get an appointment for surgery.

For the majority, the waiting list for the surgical procedure creates anguish and anxiety due to fear of bariatric surgery, weight gain, and risk of worsening health and physical limitations. In this context, the participants expect, after the bariatric surgery, to keep on being treated at the Basic Health Unit by trained professionals with a view to meeting their health needs.
